# On Hernial Protrusion

**Published:** 1824-11

**Authors:** 


### Art. III.—*On Hernial Protrusion.* By Dr. Kinglake.

A protrusion of portions of the intestines and omentum separately and conjointly, is a morbid occurrence sufficiently frequent and important to require watchful and seasonable attention. Strongly pressed as the contents of the abdomen are against its parietes under violent muscular exertion, much risk must necessarily exist on these occasions, of the more yielding parts of the boundary giving way to such protruding force. When it is considered that the regions occupied by the visceral organs are so completely filled as not to leave the smallest vacant space that could be justly regarded as a positive cavity, it is easy to perceive the extent of liability attaching to parts so compressed, for being occasionally forced beyond the natural limits so as to produce hernial disease.

It is a fact no less true than interesting, that the animal frame is universally constructed of what may be considered as mathematical cavities; but that the exigencies of the living state fill up and obliterate every area or hollow throughout the whole system, giving tone and solidity to every part of the fabric. It is obvious that the functions of life could not be performed without this universal contact of surfaces, by which the contiguity and mutual slidings of membranes are furnished that are essential to animal existence. It is this animal plenitude that subjects more particularly those parts of the abdominal viscera that are situated near the apertures that have been naturally provided as necessary outlets from the abdomen, to be casually forced in a line parallel to these openings beyond the bounds of that cavity. Seeing therefore the physical aptitude for hernial occurrences in the natural structure and functional conditions of life, it is an object of importance to be possessed of adequate means for relieving those ailments under whatever variety they may be presented.

The varieties of hernial disease have been usually comprised and enumerated under the several denominations of inguinal, scrotal, femoral, and umbilical, referring to the different situations in which they respectively appear. The prolapsed part in the two former, descends through the same aperture, taking a higher or lower position in the same channel. The femoral hernia is constituted by the protruded part passing under Poupart's ligament, in a line with the femoral artery, and other vessels proceeding from the cavity of the abdomen along the thigh in that direction. The umbilical variety of hernia is a protrusion of intestine or omentum, or both, by the side of the umbilicus or navel, formed by obliterated fœtal vessels, through which a vital communication was held before birth with the circulating fluids of the mother.

In all those various situations, a portion of intestine, with or without omentum, may by violent exertion of the abdominal muscles be extruded beyond the cavity of the belly, and may there, by the gradual enlargement of the prolapsed part, become painfully compressed, dangerously inflamed, and destructively gangrened. In intestinal hernia the obstruction to the passage of the contents of the bowels through the area of the prolapsed part may be so complete as almost necessarily to destroy life, either by the permanency of the obstacle to fœcal discharges, or what is more common, the production of sphacelus.

The danger of life in all the hernial varieties is considerable, but more especially imminent in those of the inguinal and scrotal description. The aperture through which the protrusion has been effected in those cases, being narrower than in the others, and the consequent constriction being more severe. But the destructive condition of the disease is induced by the relative size of the protruded part with that of the opening through which it has passed, occasioning a proportionately greater or less difficulty in returning it into the cavity of the belly.

As hernia in every variety is constituted by a prolapsed portion of intestine or omentum separately or conjointly, through a tendinous and an unyielding opening, the attendant danger must in every instance be estimated by the bulk of the extruded part, and the severity of the stricture by which it is confined. In intestinal hernia, the size is chiefly made up of air, the rarefaction of which causes a distending expansion that is often acutely painful, and which greatly augments the difficulty and eventual uncertainty of the case. No time should be lost, under these formidable circumstances, in endeavouring to return the hernial protrusion into the cavity of the abdomen. This is the grand curative object, to the accomplishment of which, all remedial means should be assiduously directed; and those which will prove most promptly effective, with the least concomitant pain, are surely the most eligible, and should be unhesitatingly preferred.

The taxis (as it has been quaintly termed) is usually subjected to trial more or less patiently and perseveringly, but unhappily with a result very far indeed from having been uniformly successful. The instances of failure are numerous, and the delay and hurtful violence arising from the protracted and fruitless endeavour to render the needful relief, are often among the most aggravated and irreparable circumstances of the case. If inflaming violence be used in addition to the natural proneness to inflammation, or to its actual existence, the united mischief inflicted on the prolapsed part must be of a very serious nature. It is in this state that the operation for liberating the hernial stricture is commonly resorted to; but the chance of success under such circumstances is too distant to be either gratifying to the operator or encouraging to the patient. The necessary relief should be afforded with the least possible delay and in the most effectual manner; which may be accomplished either by speedily replacing the hernial protrusion by the hand or by dividing the abdominal ring or dilating the aperture sufficiently to at once disengage the confined part and to admit of returning it securely into the cavity of the abdomen.

The mode of rendering the assistance, which it is the object of this paper to recommend, is that which has succeeded in six instances out of seven, and that too within ten minutes after commencing the trial. The principle of the treatment is founded on a leading and characteristic feature of the disease and of the practical difficulty with which its remedial means are closely connected: it is that of the contained air in the protruded intestine inflating and distending the prolapsed part to an extent that renders it too bulky to admit of its being returned through the unyielding or undilatable opening by which it has passed. What therefore should be attempted? Most certainly to diminish the flatulent distention or bulk as much as possible, so as to bring it within the area of the opening through which it is to be replaced.

This great remedial object may be effected by reduced temperature, topically applied through the medium of the coldest water that can be procured. Its sudden application by means of cloths dipped into that fluid, and renewed every two or three minutes during about half an hour will have the effect of so far abstracting heat from the hernial parts as to cause a perceptible diminution of the swelling. This is produced by the condensing influence of cold on the rarefied state of the air contained in the prolapsed portion of intestine.

When this diminution has been produced, the endeavour should be commenced to return the hernial tumor, and the mode of attempting it should be by pressing steadily but forcibly at the basis of the swelling, with a view to insinuate as it were a small portion of the air lying nearest to the hernial aperture in the first instance. This incipient step to ultimate success may with suitable management be soon effected, when the remainder will follow with the containing portion of intestine, and in passing the aperture will enter the cavity of the abdomen often with an audible report, and always with a jerk, not less satisfactory to the practitioner than gratifying to the patient. In all the instances in which this mode of relief has been pursued no subsequent inconvenience occurred; the bowels soon became with the aid of aperient medicine sufficiently active; the previous vomiting ceased; and complete recovery was the happy result.

Four of these cases were inguinal hernias, one scrotal, and the other umbilical: in each the same treatment was equally availing; and such was its efficacy as to leave no reasonable ground to doubt but equal success would with similar management attend the practice of others, and prove it to be an adequate remedy for a very afflicting and most menacing disease, with the ulterior practical advantage, that if it should not succeed, it would rather have improved than injured the prolapsed part for being ultimately liberated by instrumental assistance. The anti-inflammant influence of topical cold here, as on numerous other important occasions, would be beneficially exerted in preventing or repressing inflammatory action and thus obviating the additional difficulty that would attend cutting into and exposing parts suffering under that morbid state of excitement.

My experience warrants me in regarding it as correct practice to persist during one hour if previous relief be not obtained in the topical application of cold water, renewing it every two or three minutes by means of cloths wrung from that fluid, and following each time of applying it with a fresh attempt to return the protruded part; when, if there be no probability of succeeding, the operation should be immediately resorted to, before a dangerous state of inflammation would be likely to supervene.

In the only case in which topical cold did not render a reduction of the hernia practicable, the operation was performed without delay, the abdominal ring dilated, and the incarcerated intestine released and restored to the cavity of the belly. The patient having suffered no unnecessary delay or unavailing violence in attempting to effect a return of the prolapsed part, rapidly recovered without an unfavourable symptom, and afterwards remained permanently well.

My observation of hernial disease has been sufficiently extensive to justify me in fully believing that a correct adoption of the practice here proposed for its relief will rescue an incalculable number of sufferers from impending danger; and if it should fail in seasonably removing the constriction, it will render the operation as a measure of dernier resort, far more safe and effectual than it usually is after the indefinite delay and inefficiency attending the prevailing mode of treatment.

The attempts that are usually made to return intestinal hernia are worse than useless in aiming to effect what is often utterly impracticable. To endeavour to force into the cavity of the abdomen through a definite opening, a bulk much too large for its dimensions, is to grapple with an impossibility. The superior efficacy of topical cold in facilitating the reduction of intestinal hernia arises from its two-fold influence in condensing the rarefied air below the stricture and imparting to it a density or resisting power that renders it capable of being wielded and pressed with a wedge-like force against the abdominal aperture. Rarefied air is too unresisting to be thus acted on; and is in that attenuated state very generally an insurmountable obstaclé to the replacement of hernia. The rationale of the practice is therefore clear and cogent enough to authorize its confident adoption.

*Taunton; July 22d,* 1824.

